# Physical exercise upregulates Irisin in extracellular vesicles

**DOI:** 10.1002/alz.085413

**Published:** 2025-01-03

**Authors:** Fernanda Guarino De Felice

**Affiliations:** ^1^ Queen’s University, Kingston, ON, Canada; D'OR Institute for Research and Education, Rio de Janeiro, Rio de Janeiro Brazil

## Abstract

**Background:**

Physical exercise improves overall brain health, cognition, and stimulates the release of extracellular vesicles (EVs) in humans. Exercise upregulates irisin, a myokine derived from fibronectin type III domain‐containing protein 5 (FNDC5) previously shown to mediate the beneficial actions of exercise on memory in mouse models of Alzheimer’s disease (AD). Here, we investigated if physical exercise upregulates EVs. We further studied if EV’s cargo includes irisin and tested whether physical exercise protocols modulate its content in EVs.

**Method:**

Individuals underwent a high intensity interval training exercise program. HIIT sessions were performed 3 times a week, over 6 weeks. Serum was collected before and the day after the last exercise session. Mice were submitted to a daily swimming exercise protocol, 5 times per week, over 5 weeks. Mice were terminated 1 hour after the last bout of exercise and plasma was collected. EVs concentration and number in humans and mice. EVs obtained from the cerebrospinal fluid (CSF) from cynomolgus and Rhesus monkeys at rest obtained by lumbar punctures were also studied. We further investigated if EV’s cargo includes irisin and tested whether physical exercise protocols modulate irisin content in EVs un mice and humans. FNDC5/Irisin in EVs was detected by Western blotting, Mass spectometry and ELISA.

**Result:**

Our results indicate that irisin is associated with serum, plasma and CSF EVs from humans, mice and monkeys. Exercise upregulated EV‐associated FNDC5/Irisin in mice and humans, but did not alter circulating EV concentration. EV‐associated FNDC5/Irisin correlated positively with BDNF levels in serum obtained from humans post‐exercise.

**Conclusion:**

These findings indicate a potential physiological role of exercise‐induced upregulation of EV‐associated irisin.

